# COVID-19: preparing for second surge and getting back on track for radiology

**DOI:** 10.1259/bjro.20200045

**Published:** 2020-12-21

**Authors:** Lionel Tim-Ee Cheng, Marta E Heilbrun, Dushyant Sahani, Bien Soo Tan, Jeffrey S. Klein

**Affiliations:** 1 Department of Diagnostic Radiology, Singapore General Hospital, Singapore, Singapore; 2 Department of Radiology, Emory University School of Medicine, Atlanta, GA, USA; 3 Department of Radiology, University of Washington, Seattle, WA, USA; 4 Department of Vascular and Interventional Radiology, Division of Radiological Sciences, Singapore General Hospital, Singapore, Singapore; 5 Department of Radiology, University of Vermont College of Medicine, Burlington, VT, USA

## Abstract

In this opinion piece derived from a webinar organized by the Radiological Society of North America and conducted in the spring of 2020 during the COVID-19 pandemic, leaders from three large North American and Asian academic radiology programs review the strategies employed at their respective institutions to address the impact of the pandemic on their departments. In the first segment, the author describes the approach taken in the radiology department at an 1800-bed Asian hospital system which focuses on the creation of capacity to accommodate over 5000 COVID-19 patients in early 2020, the sustaining of services during the surge, and the development of adaptive mechanisms to address future surges and pandemics. In the second segment, a large southwestern medical system addresses the creation of a long-term strategy to provide imaging services safely for staff and patients while simultaneously utilizing technology to maintain interprofessional connections. The final segment describes how a large multifacility health-care enterprise in the Pacific Northwest of the United States is developing strategies to successfully reemerge from the forced reduction in imaging services experienced during the COVID-19 surge in early 2020.

## Introduction

Recovery and short- and long-terms strategic growth for radiology diagnostic and procedural services with COVID-19 in our communities and care facilities is a challenge for all radiology practices. Adapting local responses to the initial surge and sharing ideas for creating safe workplaces will enable radiology practices throughout the world to maintain critical patient access to the services Radiology departments and Radiologists provide. This manuscript builds on a webinar held on June 19, 2020 in which radiology leaders from Singapore and the United States share their COVID-19 experiences and provide strategies to address moving forward with persistent and new surges of COVID-19.

### Second surge and beyond: from survive to thrive

Singapore General Hospital is the largest hospital in Singapore with approximately 1800 beds, diagnostic, vascular/interventional, and nuclear medicine departments and a staff of over 700. As of mid-June 2020, our system has cared for over 5000 COVID-19 patients. Previous experience with SARS has shaped our preparation for subsequent challenges.^1,2^ We have employed three broad strategies for addressing the second surge and beyond: (1) build, (2) sustain and (3) adapt.

**Figure 1.**
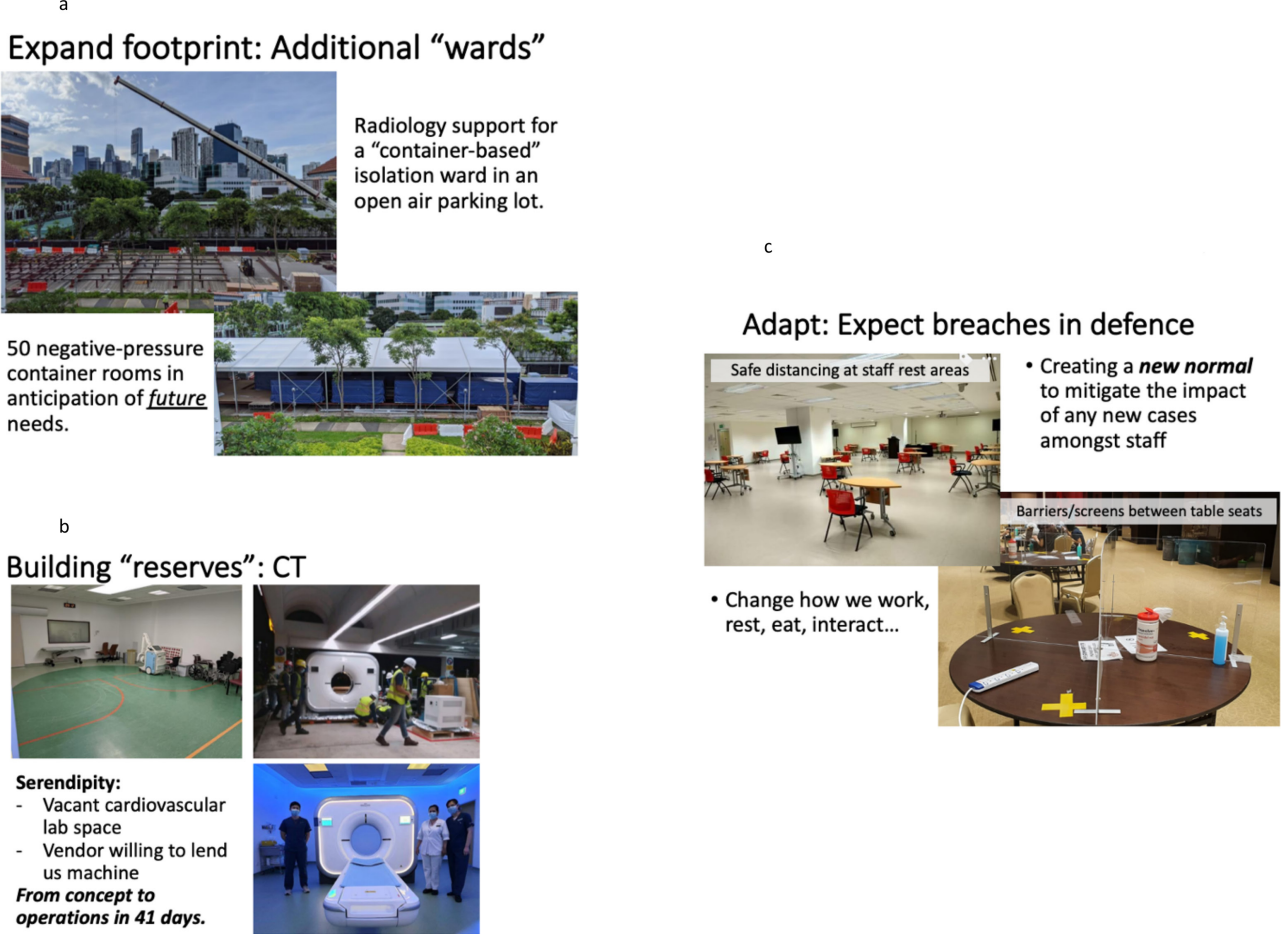
(a) Expanded hospital bed capacity for COVID-19 patients: Singapore General Hospital. (b) Building additional CT scan capacity: Singapore General Hospital. (c) Social distancing efforts in work break rooms and lunchrooms: Singapore General Hospital.

The first strategy is to “build” or create capacity before it is needed, including expanding the department footprint, securing more equipment and manpower, increasing operating hours and creating new workflows. For example, during the first surge, our emergency department expanded into an adjacent surgery center, under a pedestrian bridge and into a multistory parking garage. Our portable radiography service also expanded in tandem, utilizing a pre-built lead-lined radiography room in the garage. More recently, we built a ward in an open-air parking lot with 50 negative-pressure container rooms for additional capacity (Figure 1a). To support growing imaging demand, our department deployed portable radiography units and used a container brought to the multistory parking garage to house additional units. CT was expanded as a result of a vacant cardiovascular lab space adjacent to our department, with a scanner lent to the department installed in slightly over a month (Figure 1b).

The next strategy, to “sustain”, includes alternating work sites and adjusting work shifts. Supplies must be preserved by being more efficient and simplifying processes and imaging protocols that were unintentionally complicated. By observing our anesthesia colleagues who devised a clever telephone booth-style swabbing station, a team of radiologists, radiographers and anesthesiologists conceptualized and operationalized a new container booth X-ray facility where the radiographer remains isolated from the patient, thereby diminishing personal protective equipment consumption rates. Safe distancing and infection control requirements reduced the throughput of our imaging centers, so we fast-tracked a more nimble appointment system originally developed for our new hospital which entails a just-in-time appointment schedule that dynamically adjusts itself. Patients receive an updated precise appointment time, so that on arrival they proceed directly to be get scanned and then leave the facility. All pre- and post-scan issues are addressed via teleconsultation.^3^

The final strategy is “adapt”, as the department will need to activate reserves, reconfigure existing approaches, and adopt new rules. As the COVID-19 protocols and dedicated equipment remain in place, staff can be reactivated to provide the necessary support on short notice. Infection prevention and control measures also continue to adapt. With optimization of our processes, downtime for room preparation and CT scanner cleaning has been reduced from 5 h to under 2 h. Adapting also requires people to work outside of their comfort zone with some residents, radiographers, radiologists, nuclear medicine physicians and nurses redeployed to the ED, COVID-19 care facilities and even external sites to support the national response. Finally, we need to adapt to breaches in prevention as some staff will inevitably become infected with COVID-19. Even something as fundamental as how staff take work breaks must change (Figure 1c).

### Creating a long-term strategy for social distancing among radiology personnel

Workplaces and workforce flow adaptions during the pandemic should be governed by attention to the best available understanding of how COVID-19 is transmitted. Instituting measures to keep staff safe is of paramount concern. At Emory University, we utilize guidance from infection prevention leaders to operationalize a culture of safety. This includes flexing work hours and locations to deliver care while trying changes to meeting mission-centric goals providing patients access to the best, most necessary and appropriate care.

To create safe spaces, radiology leaders must understand how COVID-19 is spread.^2^ The current understanding is that an infected person transmits live virus when in close proximity (within 6 feet) to an uninfected person. As the infected person coughs or sneezes, respiratory droplets containing the virus land in the mouths, noses or possibly the eyes of those nearby. Many of our workspaces, including reading rooms, modality rooms, control areas and interventional radiology suites are not conducive to proper distancing.

**Figure 2.**
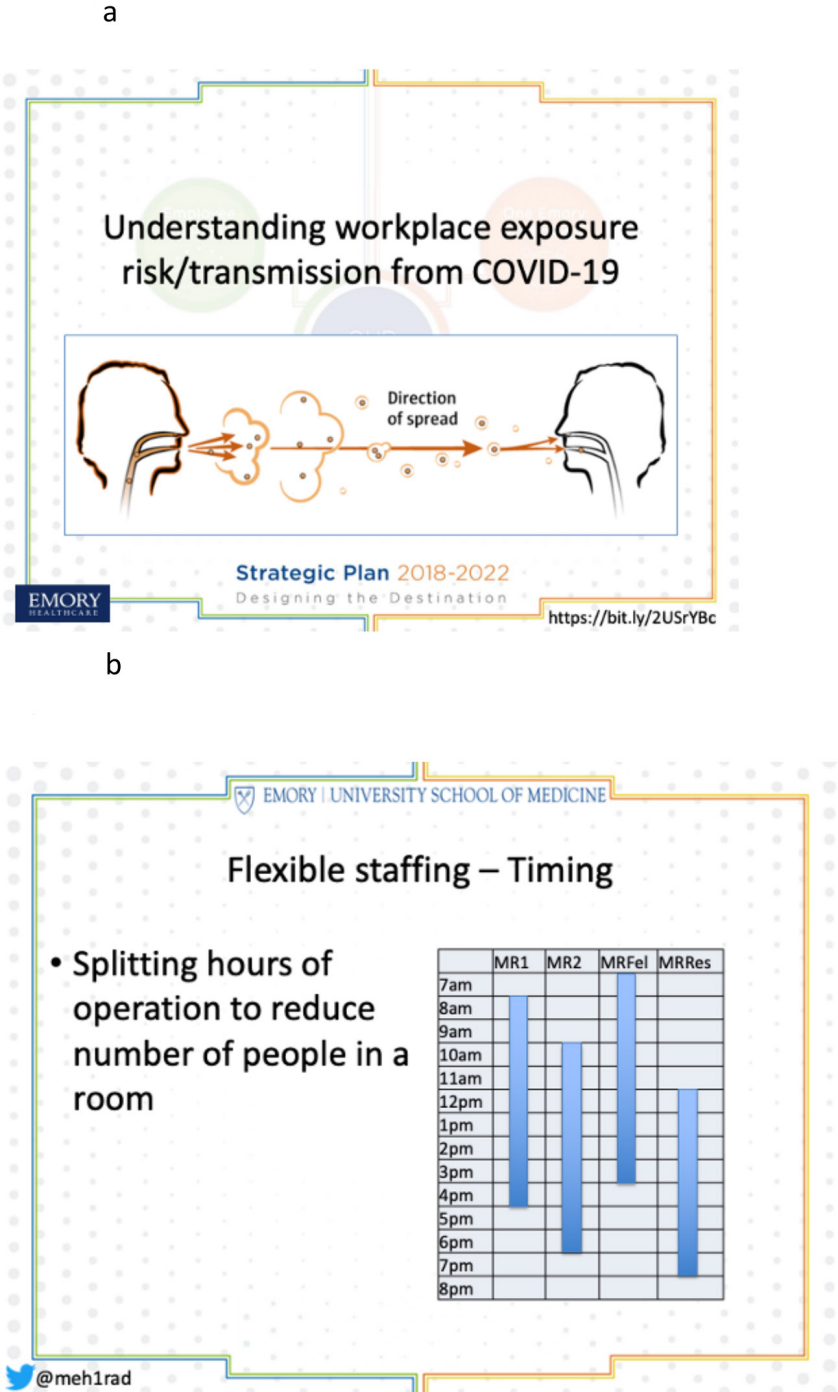
(a) Mode of COVID-19 transmission. (b) Flexible staffing. Attending and trainee shifts on MR in abdominal division at Emory University.

Dispersion of temporal and spatial image interpretation ensures a safe work environment.^4^ Workspaces can be reconfigured by moving staff and trainees off-site and separating workstations in current workrooms to provide continuous care with fewer people in any given space (Figure 2a). Rapid deployment of faculty home workstations, remote access to on-site PACS stations, and utilization of screen sharing tools allow for continuity of patient care, physical distancing, and teaching.^5^ Some sites, like the University of Wisconsin and Mayo Clinic Scottsdale, also deployed trainee home workstations. In addition to promoting physical distancing, flexible work hours and staggered shifts (Figure 2b) provide an opportunity for radiologists to reset expectations for availability of future radiologists.^6,7^

Measures that monitor workforce health also must be implemented. Staff and patients want assurances that the necessary precautions are being taken, including temperature checks and screening at facility entrances and use of online tools to check for COVID-19 symptoms. Health-care workers should model best behavior by correctly wearing masks in all locations where proper distancing is not feasible.

Physical distancing does create challenges to maintaining personal connections with colleagues, trainees, staff and patients. Online platforms can maintain a sense of community. During the initial phase of COVID-19, when in-house trainee attendance was restricted, our Emory residents participated in online remote readouts and case conferences. In this platform, multiple residents were able to see to the same material simultaneously, with more residents reviewing emergency cases. Similarly, we moved to online multidisciplinary patient care conferences which facilitated greater participation from regional physicians. Electronic tools such as Microsoft Teams™ for daily check-ins have emphasized use of social networking. Most of our training programs recently conducted virtual graduations and many divisions in our department have held social Zoom™ cocktail parties.

The adoption of electronic tools to facilitate opportunities for direct connections between radiologists, other practitioners and patients will be important as telehealth is adopted in response to COVID-19. Organizations and the trainees working in those organizations that adopt a flexible and patient-centered approach to care will realize long-term improvements in care and associated increases in professional satisfaction.

### Planning clinical strategies to get care back on track

As departments plan for recovery, they must address the disruption resulting from COVID-19. The existing backlog of deferred elective imaging procedures needs to be considered, with anticipation of the demand for imaging services and requirements for previously planned growth opportunities. Neglecting these may negatively impact care delivery, health outcomes and cause leakage of patients from the health-care system.

**Figure 3.**
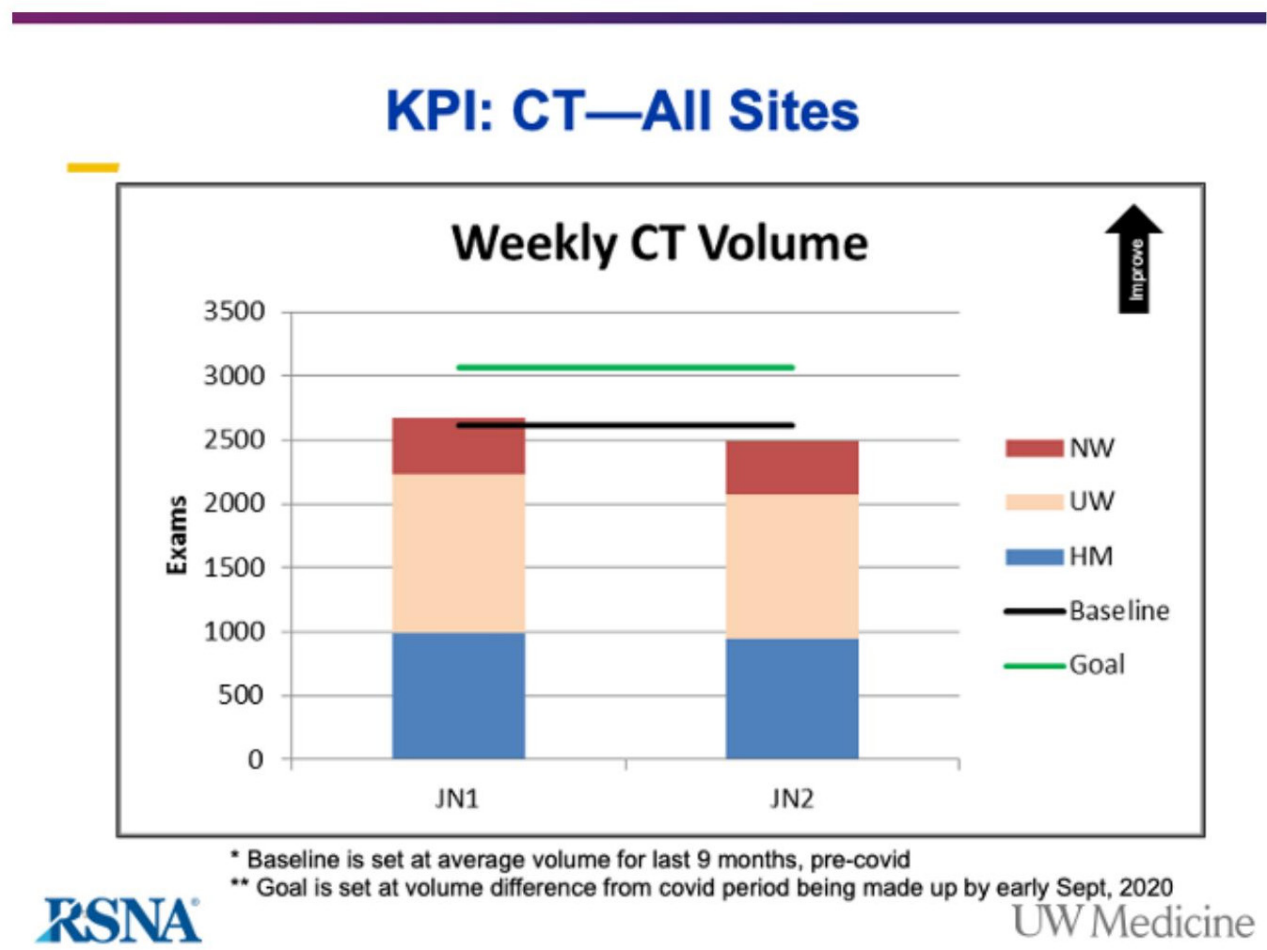
KPI for CT scans at all University of Washington Department of Radiology sites. KPI, key performance indicator.

Recovery of imaging services should ensure adequate capacity to accommodate the surge in studies during resumption of normal operations. Disruption of imaging operations caused by COVID-19 also provides an opportunity to redesign the key processes involved in scheduling and providing imaging services. It is essential to provide staff and providers with discrete goals for scheduling based on backlog and target volumes. Although it is crucial to build efficiencies in imaging workflow, we also need to increase capacity for services by considering expanded weekday and even weekend operation hours. Information technology support is required to deploy efficient operational metrics, dashboards, and data analytic tools. Departments must offer centralized scheduling with access to imaging modalities at each site of service. Concurrently, new imaging workflows should be validated by tracking key performance indicators (KPIs) (Figure 3).

**Figure 4.**
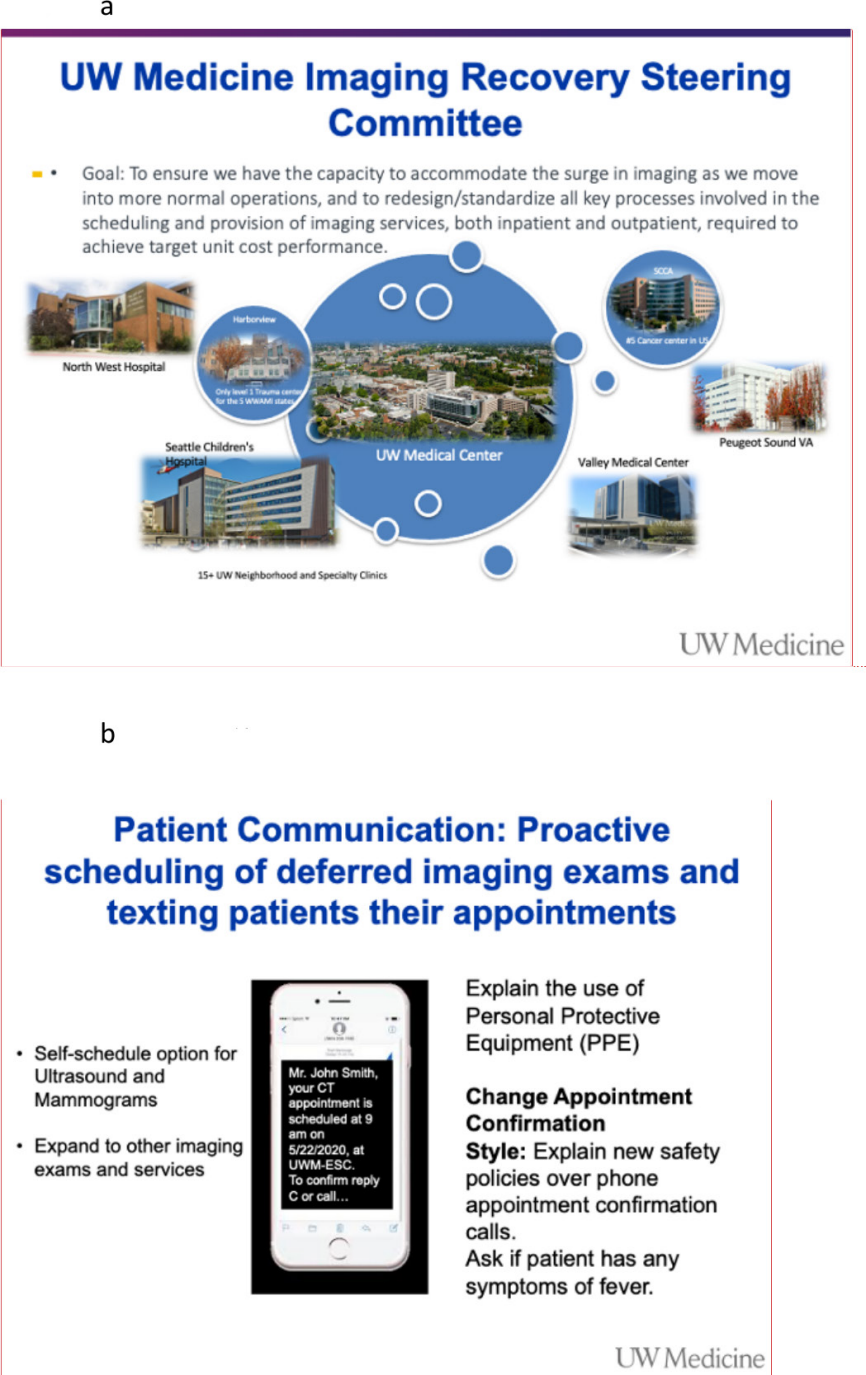
(a) University of Washington Medicine Imaging Recovery Steering Committee. (b) Text messaging with instructions to patient scheduled for imaging exam. PPE, personal protective equipment

Post-COVID-19 recovery requires a thoughtful plan to gradually resume imaging operations (Figure 4a). We deployed proactive scheduling and shared information with deferred and new exam patients via customized texting/email instructions including proper personal protective equipment use (Figure 4b). We have successfully rolled out an online scheduling option for patients to self-schedule select imaging exams, starting with routine ultrasound studies and screening mammography and then expanded to CT and MRI. On the day of service, patients receive text notifications indicating our readiness to perform examinations, thereby minimizing their time spent in facilities. In addition, appointments are staggered to reduce the number of patients simultaneously in the imaging waiting areas. To regain patients’ trust in our efforts, visible safety measures have been deployed such as ample signage to communicate the precautions being taken to keep everyone safe, mandatory masking of patients and health-care workers and ample availability of hand sanitizer. We also restricted entry to our facilities to the patient and one family member. In undergoing most interventional radiology procedures and any aerosol-generating imaging procedures such as nasogastric tube placement and modified barium swallows, we demand RT-PCR testing.

While radiology offers a broad array of imaging services, we have been strategic about our approach and focus. We established a steering committee to foster a strong team culture and alignment of imaging recovery efforts across the health system. Modality specific teams were deployed for top four imaging modalities and interventional radiology service, each comprised of a physician with domain expertise, operational lead, technologist, scheduler, and information technology personnel with representation from all service sites. While addressing the challenges from the pandemic, opportunities to build efficiencies in imaging workflow have been explored and kaizen ideas been proposed. Clustered scheduling, self-scheduling, and shorter MR protocols are being operationalized to optimize MRI capacity for improving access. Similarly, offering site-specific exam scheduling for screening exams and elective imaging studies at ambulatory sites can maximize efficiency, help standardize processes, and optimize quality while minimizing safety concerns. Reducing patient no-shows, same-day cancellations and leakage to outside facilities can ameliorate the challenges for meeting demand.

Finally, organizations should explore select long-term opportunities to lower cost, improve access and optimize imaging workflow by investing in advanced information technology solutions and automations. Consolidation of imaging and interventional services and expanding hours of clinical services is a needed measure to lower cost and improve patient satisfaction. Departments should consider investing in mobile solutions for screening mammograms, ultrasound, CT and MRI based on patient referral, service needs and geography.

### Considerations for future planning to address COVID-19 and other pandemics

- Build capacity, sustain operations, and adapt to future pandemics.
- Create safe workspaces, remote workstations and flexible work hours to provide care.
- Take precautions including monitoring of staff and physical distancing to protect staff and patients.
- Employ online technology to maintain personal connections between colleagues, staff and patients.
- Redesign key processes to restore imaging services.
- Utilize information technology to provide operational metrics and dashboards and to track key performance indicators.
- Maximize safety and efficiency of radiology appointments using online scheduling with texting and e-mail for patient communication.
- Deploy teams to optimize modality and site-specific services.
- Explore opportunities to lower cost, improve access and optimize imaging services long term.
